# A pharmacist check of patients’ infection-related condition prior to drug preparation reduces anticancer drug wastage after mixing: a retrospective study

**DOI:** 10.1186/s40545-023-00518-3

**Published:** 2023-01-17

**Authors:** Hirotsugu Yamada, Yuto Yamada, Hirotoshi Iihara, Ryo Kobayashi, Hiroyuki Tanaka, Akio Suzuki

**Affiliations:** 1 Department of Pharmacy, Gifu University Hospital, 1-1 Yanagido, Gifu, 501-1194 Japan; 2 United Graduate School of Drug Discovery and Medical Information Sciences, Gifu University, Gifu, Japan; 3 Laboratory of Advanced Medical Pharmacy, Gifu Pharmaceutical University, Gifu, Japan; 4 Laboratory of Immunobiology, Department of Biofunctional Analysis, Gifu Pharmaceutical University, Gifu, Japan

**Keywords:** Anticancer drug, Drug wastage, Cost, Pharmacist, Infection

## Abstract

**Background:**

We previously reported that a standardized pharmacist check of medical orders related to the administration criteria of anticancer drugs prior to preparation of injectable anticancer drugs was useful for reducing drug wastage after mixing. To further reduce anticancer drug wastage after preparation, we added a pharmacist check of patients' infection-related condition to the previous protocol and assessed the effectiveness of the modified protocol for reducing injectable anticancer drug wastage.

**Methods:**

In addition to the administration criteria of anticancer drugs, patients’ infection-related condition, which was based on a body temperature ≥ 37.5 °C or elevated C-reactive protein (CRP) or white blood cell (WBC) count from baseline, was added to pharmacists’ checklist of items used previously to prepare injectable anticancer drugs. We retrospectively compared the number, type and cost of anticancer drugs discarded after preparation and the reasons for discarding these drugs between pre- and post-protocol modification.

**Results:**

The rate at which anticancer drugs were discarded after preparation was significantly reduced after introducing the modified protocol compared to the original protocol (0.288% [18/6253] vs. 0.095% [6/6331], *P* = 0.013). Furthermore, the number of cases for which mixed anticancer agents were discarded because of infection decreased from 11 (fever: *n* = 8; elevated CRP or WBC: *n* = 3) to one (elevated CRP: *n* = 1) a year.

**Conclusions:**

In addition to the standard administration criteria of anticancer drugs, checking patients’ infection-related condition, defined by a body temperature ≥ 37.5 °C or elevated CRP or WBC from baseline, before mixing by the pharmacist is useful for reducing anticancer drug wastage after preparation.

## Background

The increasing cost of anticancer drugs due to the development of new agents such as molecularly targeted drugs and immune checkpoint inhibitors is a growing problem globally [1, 2]. Measures to counter drug wastage are important cost containment strategies for anticancer drugs that do not affect quality of care [3].

One of the most common causes of anticancer drug wastage is the discarding of residual drugs after preparation. As the dosage of anticancer drugs is determined based on each patient’s body weight or body surface area (BSA), there is large interpatient variability associated with dosing, and it can be difficult to completely use up the drug in a vial [4]. Drug vial optimization (DVO), which allows the use of single-use vials for multiple patients, is an effective measure for minimizing leftover anticancer drugs [5, 6]. In addition, rounding drug doses to the nearest vial size, if the difference is less than an established percentage, is also an important measure that can be implemented to minimize drug waste [7–10].

Another cause of anticancer drug wastage is the discarding of mixed anticancer drugs. Most anticancer drugs have eligibility, start of treatment, dose reduction and discontinuation criteria. Thus, dose adjustment and withdrawal of anticancer drugs must be based on patients’ bone marrow, renal, and liver function and the occurrence of side effects. If a decision to adjust the dosage or discontinue administration of anticancer drugs is made after mixing the necessary agents, the mixed anticancer drugs must be discarded. We previously reported that a pharmacist check of the eligibility, start of treatment, dose reduction and discontinuation criteria for anticancer drugs based on a standardized protocol before the mixing of injectable anticancer drugs was useful for reducing drug wastage after mixing [11].

However, patients with cancer have a high likelihood of developing infections, because treatment-related factors such as chemotherapy and progression of the tumor can increase the risk of infection [12]. Taha et al. reported that fever or infection is the second most common cause, after neutropenia, of chemotherapy delay/cancellation [13]. Ang et al. also demonstrated in a retrospective study that the most common reason for withholding chemotherapy regimens is the presence of signs and symptoms of infection, such as fever [14]. Daily monitoring of infection in patients receiving chemotherapy is, therefore, important. The criteria for body temperature at the start of treatment with anticancer drugs is typically < 38 °C in the past 24 h. Our previous standardized pharmacist check protocol was implemented based on this criterion for body temperature [11]. However, when implementing our protocol, we encountered cases with a body temperature ≥ 37.5 °C but < 38 °C for whom the planned anticancer drug dose was discontinued on the day of administration because of concerns related to infection. In these cases, the compounded anticancer drugs had to be discarded if the decision for discontinuation was made after preparation. To further reduce anticancer drug wastage after mixing, it may, therefore, be important for pharmacists to check patients’ infection-related condition, in addition to the administration criteria of anticancer drugs determined in our previous protocol.

The aim of this study was to assess the usefulness of adding a check of patients’ infection-related condition, based on a body temperature ≥ 37.5 °C or elevated C-reactive protein (CRP) or white blood cell (WBC) count from baseline, to our previous pharmacist check protocol on anticancer drug wastage. Pharmacists checked these items before mixing anticancer drugs. We compared the effectiveness of the modified and original protocols for reducing injectable anticancer drug wastage.

## Methods

### Outline of the problem and modification of the protocol

Through implementing our previous protocol, in which pharmacists checked the eligibility, start of treatment, dose reduction and discontinuation criteria for injectable anticancer drugs prior to mixing, we were able to significantly reduce anticancer drug wastage in our hospital [11]. In some cases, however, patients with a body temperature < 38 °C were identified by medical staff as having possible infection after anticancer agents had already been mixed, leading administration of anticancer drugs for the day to be discontinued. In these cases, the compounded anticancer drugs were discarded.

To eliminate such unnecessary discarding of anticancer drugs after mixing, we decided to modify the protocol. The new protocol includes a check of patients’ infection-related condition based on a body temperature ≥ 37.5 °C or elevated CRP or WBC from baseline. As in the previous protocol, pharmacists checked the criteria prior to mixing in all in-patients who received injectable anticancer agents except those who received anticancer agents that were not mixed by pharmacists. If the blood test results did not meet the criteria, the pharmacist recommended a dose change or withdrawal of anticancer drugs to the relevant physician based on the present protocol. Moreover, if the body temperature was ≥ 37.5 °C or CRP or WBC increased from baseline, the pharmacist recommended that the physician examine the patient for an infectious disease.

### Study design and study setting

This study was a single-center, retrospective chart review conducted at the 614-bed Gifu University Hospital. In-patients who received injectable anticancer agents from April 1, 2019 to March 31, 2020 (before protocol modification) and from April 1, 2020 to March 31, 2021 (after protocol modification) were enrolled in this study. Patients who received anticancer agents that were not mixed by pharmacists were excluded.

**Fig. 1:**
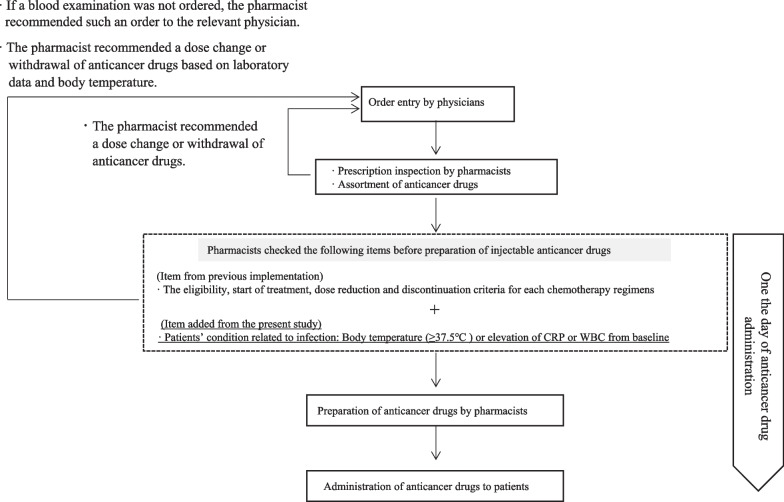
Workflow of the prescription, preparation and administration process performed by the pharmacist in charge of preparing injectable drugs

Before protocol modification, pharmacists checked the eligibility, start of treatment, dose reduction and discontinuation criteria for injectable anticancer drugs based on the protocol prior to mixing, as reported in our previous report [11]. After protocol modification, pharmacists additionally checked for a body temperature ≥ 37.5 °C or elevated CRP or WBC count from baseline (Fig. 1). All in-patients who received injectable anticancer agents, except those who received anticancer agents that were not mixed by pharmacists, were checked prior to preparation of injectable anticancer drugs. If a blood examination was not ordered, the pharmacist recommended such an order to the relevant physician. The pharmacist recommended a dose change or withdrawal of anticancer drugs based on each patient’s laboratory data and body temperature.

We recorded the number and type of anticancer drugs, contents of pharmacist interventions performed prior to mixing injectable anticancer agents, the number of anticancer drugs discarded after mixing and the reason for discarding the drugs in our dedicated database. We compared the findings between pre- and post-protocol modification.

### Evaluation of the usefulness of the modified protocol

Specifically, we compared the number and total cost of anticancer agents discarded after mixing between pre- and post-protocol modification. The cost of the discarded anticancer agents was calculated based on the drug price in Japan at the time of drug disposal. Chemotherapy cycle and chemotherapy regimen were defined based on the National Cancer Institute Dictionary of Cancer Terms, which states that a chemotherapy regimen is a treatment plan that specifies the dosage, schedule and duration of treatment with anticancer drugs, and a chemotherapy cycle is a period of treatment followed by a period of rest (no treatment) that is repeated on a regular schedule.

### Statistical analyses

Data were analyzed using IBM SPSS version 22 (IBM Japan Ltd., Tokyo, Japan). *P* values less than 0.05 were considered significant. Comparison of the total number of chemotherapy cycles and the number of mixed anticancer drugs discarded between pre- and post-protocol modification was performed using the chi**-**squared test.

### Ethics statement

This study was conducted in accordance with the guidelines for human studies adopted by the ethics committee of Gifu University Graduate School of Medicine, and notified by the Japanese government (institutional review board approval no. 2021-A062). In view of the retrospective nature of the study, subject informed consent was not required.

## Results

### Patient demographics

During the study period, patients received a total of 6253 and 6331 chemotherapy cycles before and after protocol modification, respectively (Table 1). The difference in chemotherapy regimens was significant between pre- and post-modification. After protocol modification, the number of chemotherapy cycles increased by more than 20% for patients with gynecologic cancer, colorectal cancer, gastric cancer, osteosarcoma, sarcoma, breast cancer and skin cancer, and decreased by more than 20% for those with hepatic cancer, biliary cancer, pancreatic cancer and brain cancer compared to before modification.

**Table 1:** Total number of chemotherapy cycles before and after protocol modification

Regimen	Before modification	After modification	*P* value
Hematological malignancy	1400	1268	< 0.001
Lung and respiratory organs cancer	812	764	
Gynecologic cancer	543	697	
Colorectal cancer	289	425	
Gastric cancer	66	91	
Esophageal cancer	1441	1254	
Hepatic, biliary and pancreatic cancer	193	153	
Head and neck, oral cancer	369	399	
Pediatric cancer	527	535	
Osteosarcoma and sarcoma	189	291	
Breast cancer	7	13	
Brain cancer	69	43	
Urological cancer	306	341	
Skin cancer	22	31	
Other cancer	20	26	
Total	6253	6331	

Data were statistically compared using the chi-squared test

### Comparison of the number of injectable anticancer drugs discarded

**Fig. 2:**
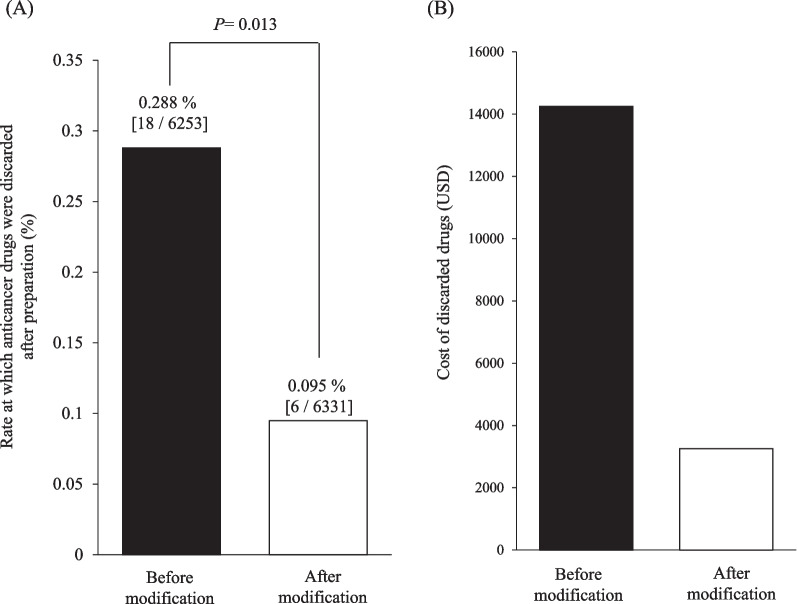
Comparison of the percentage (**A**) and cost (**B**) of injectable anticancer drugs discarded after preparation between pre- and post-protocol modification. Data were statistically compared using the chi-squared test

After protocol modification, the rate at which anticancer drugs in chemotherapy cycles were discarded after preparation was significantly reduced compared with that before protocol modification (0.288% [18/6253] vs. 0.095% [6/6331], *P* = 0.013) (Fig. 2A). The total cost of the discarded compounded anticancer drugs was reduced from USD 14,247 before modification to USD 3252 after modification (Fig. 2B).

The most frequent reason for discarding mixed anticancer agents before protocol modification was fever (*n* = 8, 44.4%), followed by elevated CRP or WBC (*n* = 3, 16.7%), myelosuppression (*n* = 2, 11.1%), patients’ request (*n* = 2, 11.1%) and other abnormalities in patients’ blood test results (*n* = 1, 0.6%) (Table 2). After protocol modification, the occurrence of drug wastage for the following four reasons dropped to just one case each: elevated CRP, myelosuppression, patients’ request and other abnormalities in patients’ blood test results. Notably, fever was no longer a reason for discarding anticancer drugs (Table 2).

**Table 2:** Reasons for discarding mixed injectable anticancer agents before and after protocol modification

Reason	Before modification (%)	After modification (%)
Patients’ infection-related condition		
Fever (≥ 37.5 °C)	8 (44.4)	0
Elevated CRP or WBC	3 (16.7)	1 (16.6)
Patients’ request	2 (11.1)	1 (16.6)
Myelosupression	2 (11.1)	1 (16.6)
Other	3 (16.7)	3 (50.2)
Total	18	6

*CRP* C-reactive protein, *WBC* white blood cell

### Contents of interventions by pharmacists before and after protocol modification

The contents of interventions by pharmacists performed prior to mixing injectable anticancer agents before and after protocol modification are shown in Table 3. The total number of interventions performed pre- and post-modification was 141 and 159, respectively. Myelosuppression was the most common intervention in both cases (116 [82.2%] before modification, 125 [78.6%] after modification). Meanwhile, the number of interventions for fever and elevated CRP or WBC increased after protocol modification (fever: 1 [0.7%] vs. 8 [5.0%], elevated CRP or WBC: 2 [1.4%] vs. 8 [5.0%]).

**Table 3:** Comparison of the contents of pharmacist interventions performed prior to mixing injectable anticancer agents before and after protocol modification

Intervention	Number of interventions (%)	
	Before modification	After modification
Patients’ infection-related condition		
Fever (≥ 37.5 °C)	1 (0.7)	8 (5.0)
Elevated CRP or WBC	2 (1.4)	8 (5.0)
Myelosupression	116 (82.3)	125 (78.6)
Renal dysfunction	10 (7.1)	4 (2.5)
Hepatic dysfunction	9 (6.4)	8 (5.0)
Electrolytic imbalance	3 (2.1)	2 (1.3)
Anemia	0	3 (2.0)
Peripheral neuropathy	0	1 (0.6)
Total	141	159

*CRP* C-reactive protein, *WBC* white blood cell

## Discussion

We found that adding a check by pharmacists of patients’ infection-related condition to a previously devised protocol, based on body temperature ≥ 37.5 °C or elevated CRP or WBC from baseline, before anticancer drug mixing significantly reduced anticancer drug wastage after mixing.

In our previous protocol, a body temperature < 38 °C was used as an administration criterion before anticancer drug mixing. However, in 11 cases per year, anticancer drugs were discarded for reasons related to infection. During the same period, pharmacists conducted just three interventions related to infection. After adding a check of patients’ infection-related condition to the previous protocol, the number of cases for which mixed anticancer agents were discarded because of infection decreased from 11 to one a year. In addition, the number of interventions related to infection conducted by pharmacists increased to 16 a year. Consequently, the number of cases in which anticancer drugs were discarded after preparation decreased from 18 cases before protocol modification to six cases afterward. Furthermore, the total cost of discarded compounded anticancer drugs dropped from USD 14,247 before modification to USD 3252 afterward. Ang et al. also reported that the total cost of parenteral cytotoxic wastage for returned chemotherapy regimens in 72 cases a year at a tertiary hospital was 2052 EUR [14]. It is important to note that the savings effect was affected by the type of anticancer drug discarded, although the average price of anticancer drugs has increased [15]. On the other hand, Shayne et al. showed in a retrospective cohort study that infection was one of the risk factors for in-hospital mortality and prolonged length of stay in older patients with cancer [16]. Thus, the protocol reported in the present study may also contribute to the improvement of patient prognosis and reduction of medical expenses by reducing patients’ length of hospital stay.

We examined patients’ infection-related condition based on a body temperature ≥ 37.5 °C or elevated CRP or WBC from baseline in this study. According to the Common Terminology Criteria for Adverse Events (CTCAE) v5.0 [17], fever is defined as a body temperature > 38 °C, and febrile neutropenia (FN), a critical complication associated with mortality in patients receiving chemotherapy, is defined as an oral temperature > 38.3 °C. The European Society for Medical Oncology (ESMO) guidelines defines FN as two consecutive readings of > 38.0 °C in 1 h [18]. The body temperature criterion for starting treatment with almost any anticancer drug is, therefore, set at < 38 °C. However, several research studies have defined ‘normal’ body temperature as < 37.5 °C, and use a body temperature ≥ 37.5 °C for diagnosing infection [19, 20]. In this study, the number of interventions conducted by pharmacists in a year increased from one to eight after we changed the criteria for body temperature from < 38.0 to < 37.5 °C, and the number of discarded mixed anticancer agents related to suspected infection decreased from eight to zero.

We also added a check for elevated CRP or WBC from baseline to the modified protocol. The number of interventions related to elevated CRP or WBC conducted by pharmacists in a year increased from two to eight, and the number of discarded mixed anticancer agents related to elevated CRP or WBC decreased from three to one. CRP level and WBC count have been used as early biomarkers of infection [21, 22]. However, it should be noted that elevated levels of these biomarkers are also observed in other inflammation reactions, especially cancer development and progression [23–25].

There were six cases in which compounded anticancer drugs were discarded after protocol modification. Of these, three cases that met the administration criteria of anticancer drugs discontinued treatment due to a decision by their physicians. In the other three cases, treatment was discontinued due to sudden changes in the patients’ condition on the day of anticancer drug administration, which is difficult to prevent.

## Study limitations

There were several limitations in the present study. First, as the study was conducted under a retrospective and non‐randomized observational design, various unknown patient selection processes may have biased the outcome. Second, we could not evaluate the burden of implementing the present measure on human resources. Finally, an analysis from the perspective of the medical safety of anticancer drug administration could not be conducted.

## Conclusions

This study showed that checking patients’ infection-related condition, in addition to the administration criteria of anticancer drugs, before mixing by the pharmacist is useful for reducing anticancer drug wastage after preparation.

## Data Availability

The data sets obtained and analyzed in the current study are available from the corresponding author on reasonable request.

## Abbreviations

DVO: Drug vial optimization
CRP: C-reactive protein
WBC: White blood cell
CTCAE: The Common Terminology Criteria for Adverse Events
FN: Febrile neutropenia
ESMO: European Society for Medical Oncology
